# Altered phenotypic and functional characteristics of CD3^+^CD56^+^ NKT-like cells in human gastric cancer

**DOI:** 10.18632/oncotarget.10484

**Published:** 2016-07-08

**Authors:** Liu-sheng Peng, Fang-yuan Mao, Yong-liang Zhao, Ting-ting Wang, Na Chen, Jin-yu Zhang, Ping Cheng, Wen-hua Li, Yi-pin Lv, Yong-sheng Teng, Gang Guo, Ping Luo, Weisan Chen, Quan-ming Zou, Yuan Zhuang

**Affiliations:** ^1^ National Engineering Research Center of Immunological Products, Department of Microbiology and Biochemical Pharmacy, College of Pharmacy, Third Military Medical University, Chongqing, PR China; ^2^ Department of General Surgery and Center of Minimal Invasive Gastrointestinal Surgery, Southwest Hospital, Third Military Medical University, Chongqing, PR China; ^3^ La Trobe Institute for Molecular Science, La Trobe University, Bundoora, Victoria, Australia

**Keywords:** NKT-like cells, gastric cancer, functional impairment, tumor progression, immune escape

## Abstract

CD3^+^CD56^+^ natural killer T (NKT)-like cells are a group of CD3^+^ T cells sharing characteristics of NK and T cells and constitute a major component of host anti-tumor immune response in human cancer. However, the nature, function and clinical relevance of CD3^+^CD56^+^ NKT-like cells in human gastric cancer (GC) remain unclear. In this study, we showed that the frequencies of CD3^+^CD56^+^NKT-like cells in GC tumors were significantly decreased and low levels of tumor-infiltrating CD3^+^CD56^+^ NKT-like cells were positively correlated with poor survival and disease progression. Most CD3^+^CD56^+^NKT-like cells in GC tumors were CD45RA^−^CD27^+/−^ central/effector-memory cells with decreased activity and lower expression levels of CD69, NKG2D and DNAM-1 than those in non-tumor tissues. We further observed that tumor-infiltrating CD3^+^CD56^+^ NKT-like cells had impaired effector function as shown by decreased IFN-γ, TNF-α, granzyme B and Ki-67 expression. Moreover, *in vitro* studies showed that soluble factors released from GC tumors could induce the functional impairment of CD3^+^CD56^+^ NKT-like cells. Collectively, our data indicate that decreased tumor-infiltrating CD3^+^CD56^+^ NKT-like cells with impaired effector function are associated with tumor progression and poor survival of GC patients, which may contribute to immune escape of GC.

## INTRODUCTION

Gastric cancer (GC) is the fourth most common cancer worldwide often with poor prognosis [1]. The clinical relevance of GC has been recognized to be influenced by the cross-talk between tumors and host immune system, and a strong anti-tumor immune response in the tumor microenvironment is associated with a favorable clinical outcome [2]. However, although immune cell infiltration in GC is often observed, the anti-tumor immunity is commonly ineffective [3, 4].

CD3^+^CD56^+^ NKT-like cells are a broad group of CD3^+^ T cells co-expressing T-cell antigen receptor (TCR) and NK-cell markers [5, 6]. That is, CD3^+^CD56^+^ NKT-like cells share NK and T cell characteristics and possess both innate and acquired immune functions. Once TCR ligation, CD3^+^CD56^+^ NKT-like cells can be activated to secrete cytotoxic enzymes and cytokines to kill target cells [7]. On the other hand, CD3^+^CD56^+^ NKT-like cells are also able to mediate non-MHC-restricted lysis and cytokine production in the absence of TCR activation. Therefore, CD3^+^CD56^+^ NKT-like cells have been postulated to play an important role in anti-tumor and anti-virus immune response [8, 9]. In human cancers, including lung and colorectal cancers, high levels of CD3^+^CD56^+^ NKT-like cells have been reported to be associated with improved patient's survival [10, 11]. However, studies focusing on the functional activity of CD3^+^CD56^+^NKT-like cells showed that the cytotoxicity and cytokine production of these cells are impaired in haematological malignancies and other solid tumors, such as chronic lymphocytic leukemia and ovarian cancer [11, 12]. Interestingly, in acute leukemia, although higher numbers of CD3^+^CD56^+^NKT-like cells were observed in the peripheral blood, their functions were significantly impaired [13]. Taken together, these data suggest that the anti-tumor activity of CD3^+^CD56^+^ NKT-like cells is likely altered in the tumor microenvironment. To our knowledge, the nature, function and clinical relevance of CD3^+^CD56^+^ NKT-like cells in GC patients remain largely unexplored.

In the present study, we firstly aim to examine the levels and functional activity of CD3^+^CD56^+^ NKT-like cells in tumor and non-tumor tissues of GC patients, and then analyze the possible associations between these cells and clinical features of GC. We show that decreased levels of tumor-infiltrating CD3^+^CD56^+^ NKT-like cells with functional impairment correlated with tumor progression and poor overall survival of GC patients. Restoring their effector function should benefit anti-tumor immunity to GC.

## RESULTS

### Frequencies and clinical associations of CD3^+^CD56^+^NKT-like cells in tumors of GC patients

**Figure 1:**
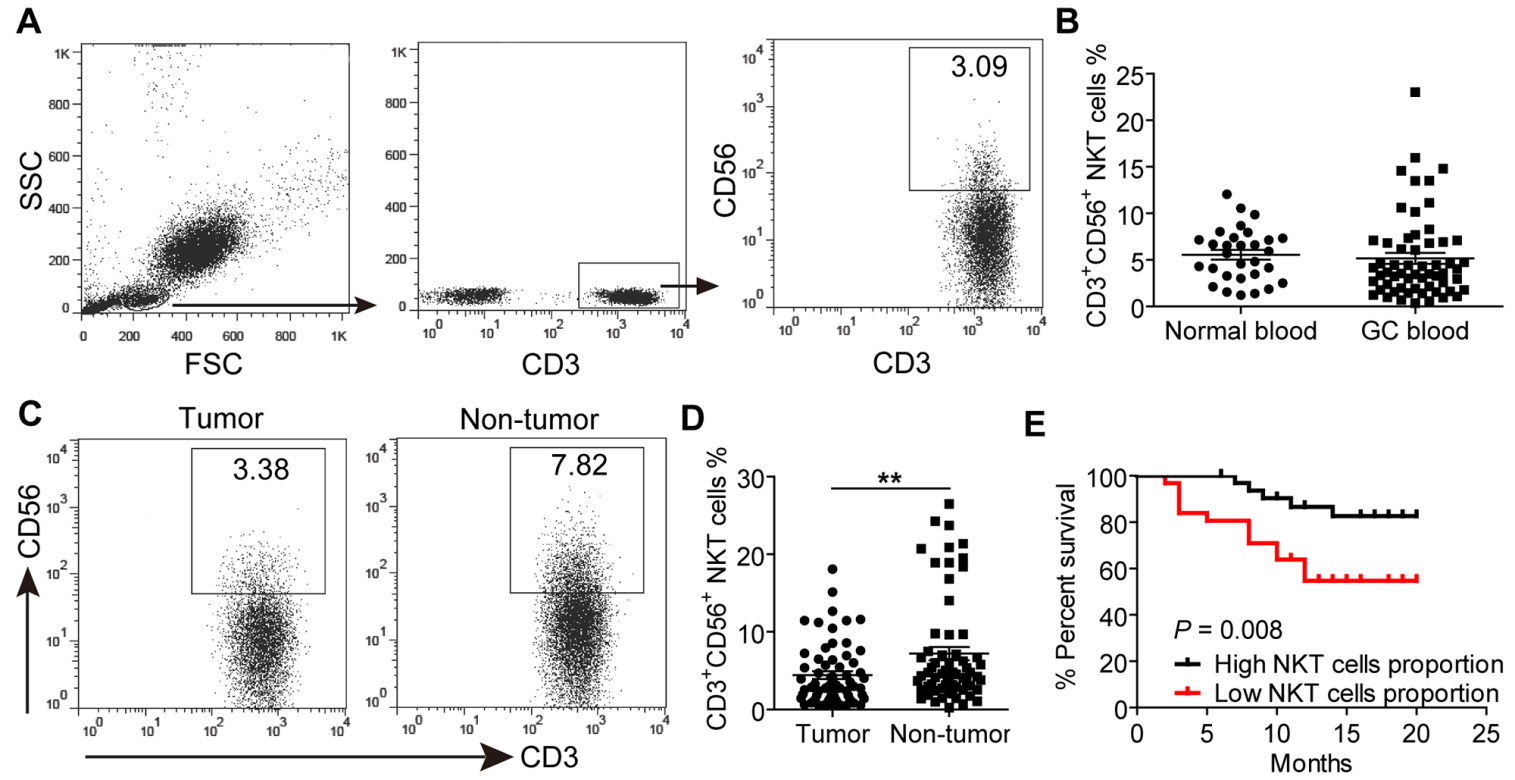
The prevalence of CD3+CD56+ NKT-like cells in peripheral blood, non-tumor and tumor tissues of GC patients **A.** A representative flow cytometry analysis of CD3^+^CD56^+^ NKT-like cells in whole peripheral blood of GC patients after gating on CD3^+^ T cells. **B.** The frequencies of CD3^+^CD56^+^ NKT-like cells in peripheral blood of 30 normal controls and 63 GC patients. **C.** The representative dot plot of CD3^+^CD56^+^ NKT-like cells after gating on CD3^+^ T cells in non-tumor and tumor tissues of the same patient. **D.** The frequencies of CD3^+^CD56^+^ NKT-like cells in CD3^+^ T cells from non-tumor and tumor tissues of 63 patients. **E.** Kaplan–Meier plots for patients' overall survival by the median of tumor-infiltrating CD3^+^CD56^+^ NKT-like cells frequency (2.99%). Statistical analysis in (B) and (D) was separately shown by unpaired and paired Student's *t* test. Data were the mean ± SEM; **P<0.01.

Using flow cytometry, we first analyzed the presence of CD3^+^CD56^+^ NKT-like cells in the peripheral blood of GC patients and healthy individuals. Figure 1A shows the gating strategy. We found that there was no difference in the frequencies of circulating CD3^+^CD56^+^ NKT-like cells between healthy individuals and GC patients (Figure 1B, 5.17%, 0.37%-23.01% vs. 5.55%, 1.22%-12.04%, *P*>0.05). Next we evaluated CD3^+^CD56^+^ NKT-like cell frequencies in non-tumor and tumor tissues of GC patients. As shown in Figure 1C and 1D, the frequencies of CD3^+^CD56^+^ NKT-like cells in tumors were significantly lower than those in non-tumor tissues (4.44%, 0.52%-18.10% vs. 7.20%, 0.23%-26.50%, *P*<0.01). Furthermore, low CD3^+^CD56^+^ NKT-like cell frequencies were shown to be positively correlated with patients' poorer overall survival according to the median value of tumor-infiltrating CD3^+^CD56^+^ NKT-like cell frequencies (Figure 1E). In addition, low levels of tumor-infiltrating CD3^+^CD56^+^ NKT-like cells were also positively correlated with advanced clinical features, including tumor size, tumor invasion, distant metastasis and tumor-node-metastasis (TNM) stages (Supplementary Figure S1). Thus, these data suggested that decreased tumor-infiltrating CD3^+^CD56^+^ NKT-like cells are associated with tumor progression and GC patients' poorer overall survival.

### Phenotypic features of CD3^+^CD56^+^NKT-like cells at tumor site

**Figure 2:**
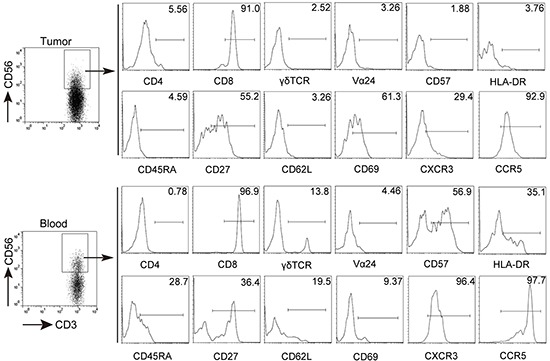
Phenotypic characteristics of CD3+CD56+ NKT-like cells in GC patients Freshly isolated cells from tumor tissues and peripheral blood were stained with anti-CD3, anti-CD56, anti-CD4, anti-CD8, anti-γδ TCR, anti-Vα 24, anti-CD57, anti-HLA-DR, anti-CD45RA, anti-CD27, anti-CD62L, anti-CD69, CXCR3 and CCR5 antibodies. Cells were gated on CD3^+^CD56^+^ NKT-like cells and the expression of CD4, CD8, γδ TCR, Vα 24, CD57, HLA-DR, CD45RA, CD27, CD62L, CD69, CXCR3, CCR5 was analyzed.

**Figure 3:**
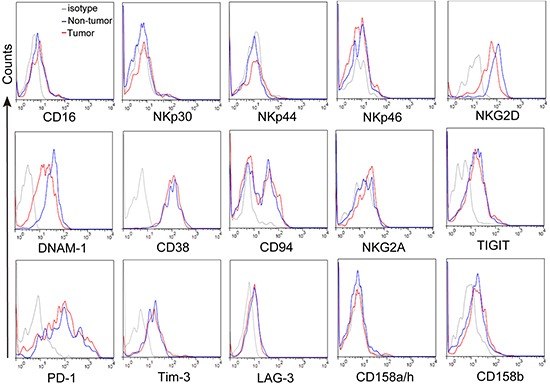
The expression of NK-cell-associated activating and inhibitory receptors on CD3+CD56+ NKT-like cells Freshly isolated cells from tumor and non-tumor tissues were stained with anti-CD3, anti-CD56, anti-CD16, anti-NKp30, anti-NKp44, anti- NKp46, anti-NKG2D, anti-DNAM-1, anti-CD38, anti-CD94, anti-NKG2A, anti-TIGIT, anti-PD-1, anti-Tim-3, anti-LAG-3, anti-CD158a/h and anti-CD158b antibodies. Cells were gated on CD3^+^CD56^+^ NKT-like cells and the expression of CD16, NKp30, NKp44, NKp46, NKG2D, DNAM-1, CD38, CD94, and NKG2A, TIGIT, PD-1, Tim-3, LAG-3, CD158a/h, CD158b was analyzed.

Next we studied the phenotypic features of CD3^+^CD56^+^ NKT-like cells at tumor site. As shown in Figure 2, we observed that the large majority of CD3^+^CD56^+^ NKT-like cells in tumor tissues belonged to the CD8^+^ subset and were nearly devoid of γδ TCR^+^ cells and invariant Vα24^+^ NKT cells. In addition, tumor-infiltrating CD3^+^CD56^+^ NKT-like cells express low levels of CD45RA but intermediate level of CD27, suggesting that these cells were CD45RA^−^CD27^+/−^ central/effector-memory cells (Supplementary Figure S2). Furthermore, we found that a higher level of CD69 on tumor-infiltrating CD3^+^CD56^+^ NKT-like cells than those on circulating CD3^+^CD56^+^ NKT-like cells. However, compared with non-tumor tissues, the level of CD69 expression on CD3^+^CD56^+^ NKT-like cells in tumor tissues was significantly decreased (Supplementary Figure S3). We also observed that CD3^+^CD56^+^ NKT-like cells in tumors expressed lower level of inflammatory tissue homing markers CXCR3 and CCR5 than those in the peripheral blood. CD3^+^CD56^+^ NKT-like cells share similar surface receptor expression with NK cells. Therefore, the expression of NK-cell-associated activating and inhibitory receptors on CD3^+^CD56^+^ NKT-like cells were determined. As shown in Figure 3, there were no differences for the expression of activating receptors including CD16, NKp30, NKp44, NKp46, CD38, CD94 and inhibitory receptors including NKG2A, TIGIT, PD-1, Tim-3, LAG-3, CD158a/h, CD158b on these cells in non-tumor and tumor tissues. However, the expression levels of activating receptors NKG2D and DNAM-1 on tumor-infiltrating CD3^+^CD56^+^ NKT-like cells were significantly lower than those on non-tumor-infiltrating these cells (Figure 3 and S4). Based on the above observations, we concluded that CD3^+^CD56^+^ NKT-like cells at tumor sites in GC patients have an overall central/effector-memory phenotype with decreased activation.

### Effector function of GC-infiltrating CD3^+^CD56^+^NKT-like cells

**Figure 4:**
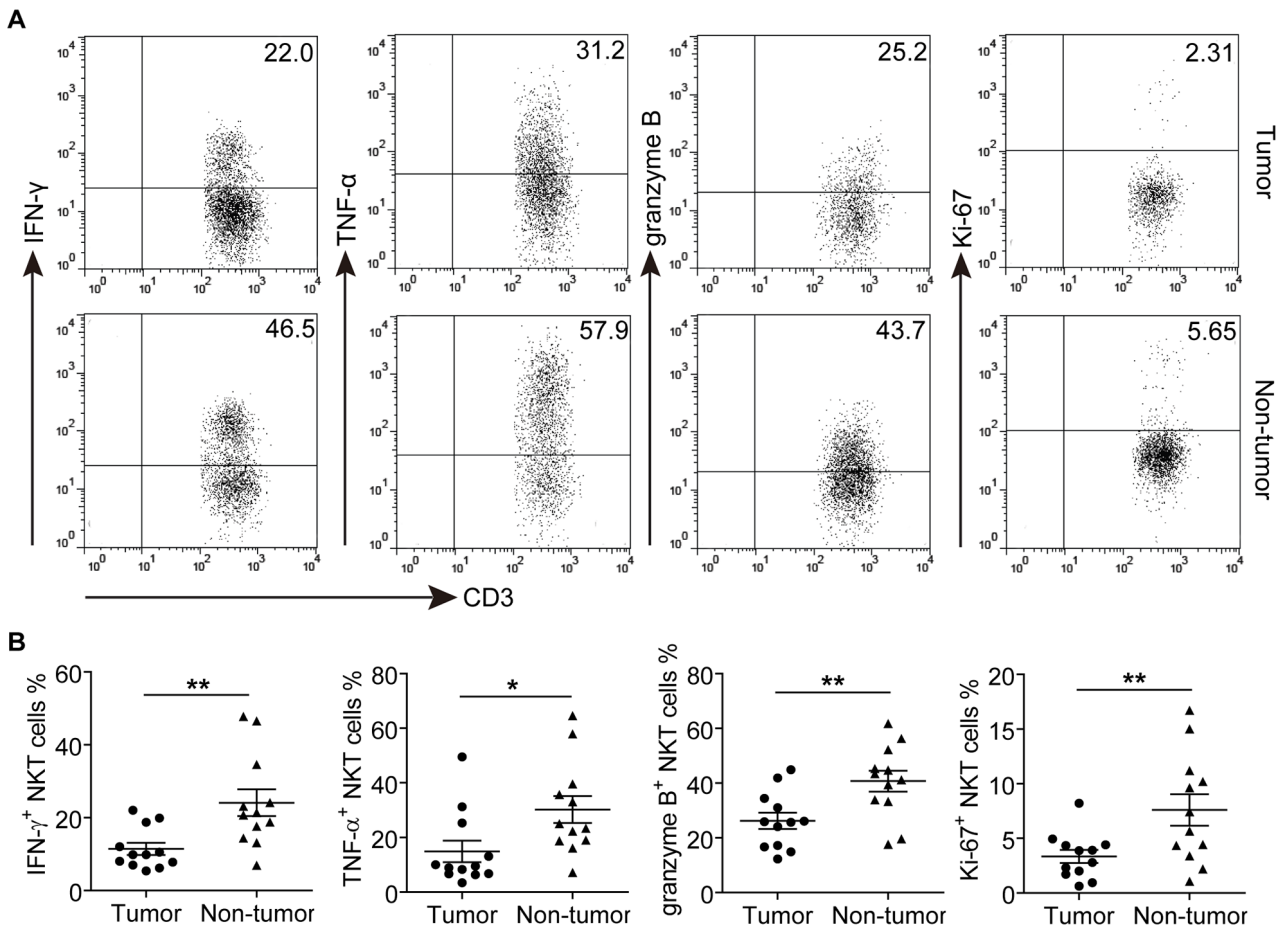
The effector functions of tumor and non-tumor-infiltrating CD3+CD56+ NKT-like cells in GC patients **A-B.** Representative dot plots (A) and Statistical analyses (B) of IFN-γ, TNF-α, granzyme B and Ki-67 in CD3^+^CD56^+^ NKT-like cells from paired tumor and non-tumor tissues of GC patient. Symbols represent individual values, and 12 GC patients analyzed individually. *P<0.05, **P<0.01.

To investigate the effector function of CD3^+^CD56^+^ NKT-like cells, polyclonal stimulation was carried out for detecting intracellular cytokines production. As shown in Figure 4, the majority of non-tumor-infiltrating CD3^+^CD56^+^ NKT-like cells produced IFN-γ and TNF-α, but in tumors, we observed that significantly lower frequencies of IFN-γ and TNF-α-producing CD3^+^CD56^+^ NKT-like cells than those in non-tumor tissues. In addition, the frequency of granzyme B in tumor-infiltrating CD3^+^CD56^+^ NKT-like cells was also lower than that of the counterparts in non-tumor tissues, suggesting that the cytokine and cytotoxic molecule production of tumor-infiltrating CD3^+^CD56^+^ NKT-like cells were decreased. We further analyzed the proliferation status of tumor-infiltrating CD3^+^CD56^+^ NKT-like cells. Although relatively low levels of proliferation-associated marker Ki-67 were detected in both non-tumor and tumor-infiltrating CD3^+^CD56^+^ NKT-like cell populations, Ki-67 level in tumor-infiltrating CD3^+^CD56^+^ NKT-like cells were significantly decreased. Taken together, these results indicated that while infiltrated in the tumors, the effector function of CD3^+^CD56^+^ NKT-like cells were likely impaired.

### Effect of tumor microenvironment on CD3^+^CD56^+^NKT-like cells' effector function

**Figure 5:**
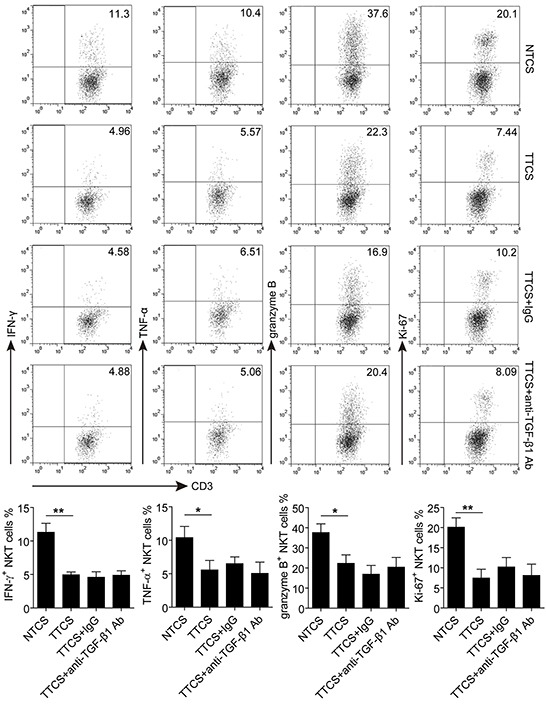
The functional impairment of CD3+CD56+ NKT-like cells induced by soluble factor(s) from tumor tissue culture supernatant PBMCs from healthy individuals were cultured for 48 hours with 10% TTCS or NTCS in the presence of rhIL-2, anti-CD3 and anti-CD28 antibodies. Where indicated, 10 μg/ml anti-TGF-β1 or isotype IgG antibody was added as described in Materials and Methods. The expression of IFN-γ, TNF-α, granzyme B and Ki-67 in CD3^+^CD56^+^ NKT-like cells were determined by flow cytometry. In total, PBMCs from three healthy individuals were used to culture with TTCS and NTCS from two GC patients. *P<0.05, **P<0.01. Ab, antibody.

To address whether soluble factors from GC microenvironment could influence the effector function of CD3^+^CD56^+^ NKT-like cells, tumor tissue culture supernatant (TTCS) and non-tumor tissue culture supernatant (NTCS) were used to culture PBMCs from healthy individuals for 48 hours. As shown in Figure 5, the expression of IFN-γ, TNF-α, granzyme B and Ki-67 in TTCS-treated CD3^+^CD56^+^ NKT-like cells were significantly lower that those in the NTCS-treated group, suggesting that the effector function of CD3^+^CD56^+^ NKT-like cells is suppressed by soluble factor from TTCS. However, such functional impairment of CD3^+^CD56^+^ NKT-like cells was independent on TGF-β1 as blocking TGF-β1 signaling did not significantly attenuate TTCS-mediated suppression of IFN-γ, TNF-α, granzyme B and Ki-67 in CD3^+^CD56^+^ NKT-like cells. Above all, these results suggested that soluble factor(s) from GC tumors could induce the functional impairment of CD3^+^CD56^+^ NKT-like cells.

## DISCUSSION

Deciphering the role of host immune system in the tumor microenvironment is crucial for understanding how they affect the development and course of human tumors [14, 15]. Despite recent success in delineating the function and clinical relevance of tumor-infiltrating lymphocytes (TIL) including conventional CD3^+^ T cells and CD3^−^CD56^+^ NK cells in human GC [16, 17], the relationship between CD3^+^CD56^+^ NKT-like cells, another subset of TIL, and GC progression is completely unknown. Here, our study showed for the first time that within GC, the frequencies of CD3^+^CD56^+^ NKT-like cells were significantly decreased, and low levels of CD3^+^CD56^+^ NKT-like cells were positively correlated with patients' poor overall survival, suggesting that the frequencies of tumor-infiltrating CD3^+^CD56^+^ NKT-like cells may be a good marker of GC patients' prognosis. Furthermore, we observed a strong negative association between their levels and advanced clinical features, including tumor size, tumor invasion, distant metastasis and TNM stages, indicating that lower level of CD3^+^CD56^+^ NKT-like cells in tumors may have contributed to GC progression.

CD3^+^CD56^+^ NKT-like cells have been reported to be highly heterogeneous, for they contain CD4^+^, CD8^+^, CD4^−^CD8^−^ and even γδ TCR^+^ cells and invariant Vα24^+^ NKT cells [18]. However, in our study, we showed that the majority of these cells in tumor tissues expressed CD8 molecule but not γδ or Vα24 NKT cell TCR, indicating that most of the GC-infiltrating CD3^+^CD56^+^ NKT-like cells were CD8^+^ T cells, not γδ T cells or invariant Vα24^+^ NKT cells. CXCR3 and CCR5 are inflammatory tissue homing molecules that play a crucial role in CD8^+^ T cell migration [19]. However, their levels on GC-infiltrating CD3^+^CD56^+^ NKT-like cells were lower than those in the peripheral blood, implying that a significant drop of CXCR3 and CCR5 expression on CD3^+^CD56^+^ NKT-like cells may be accompanied by the infiltration of these cells in tumors. The cell differentiation and activation status is important for their effector function [20, 21]. The combination CD45RA and CD27 expression can separate human T cells into four subsets: CD45RA^+^CD27^+^ naïve, CD45RA^−^CD27^+^ central-memory, CD45RA^−^CD27^−^ effector-memory and CD45RA^+^CD27^−^ terminally-differentiated T cells [22, 23]. We observed that tumor-infiltrating CD3^+^CD56^+^ NKT-like cells belonged to the CD45RA^−^CD27^+^ and CD45RA^−^CD27^−^ subset, indicating that these CD3^+^CD56^+^ NKT-like cells were central/effector-memory cells and matured in tumors. However, the activation marker CD69 expressing on CD3^+^CD56^+^ NKT-like cells in tumors were significantly fewer than those in non-tumor tissues. Furthermore, NK-cell activating receptors NKG2D and DNAM-1 expression were also decreased on tumor-infiltrating CD3^+^CD56^+^ NKT-like cells. Therefore, although CD3^+^CD56^+^ NKT-like cells in GC tumors were shown to have a matured phenotype, their activities might be suppressed. CD3^+^CD56^+^ NKT-like cells carry out their anti-tumor function by producing cytokines and/or releasing cytotoxic molecules [24]. However, our study showed that CD3^+^CD56^+^ NKT-like cells in GC tumors displayed impaired effector function when stimulated, such as lower IFN-γ and TNF-α production than those in non-tumor tissues. Moreover, the expression of granzyme B and proliferation-associated marker Ki-67 in tumor-infiltrating CD3^+^CD56^+^ NKT-like cells were also decreased. Therefore, decreased cytokines production, cytotoxic potential and proliferation capacity of CD3^+^CD56^+^ NKT-like cells will likely lead to worsening clinical course.

It is important to elucidate the functional regulation of CD3^+^CD56^+^ NKT-like cells in GC. Co-inhibitory molecules such as PD-1, Tim-3, LAG-3 and TIGIT have been widely reported to inhibit T-cell and NK-cell function [25–27]. However, the expression of these molecules were not altered on tumor-infiltrating CD3^+^CD56^+^ NKT-like cells from GC patients. Instead, soluble inhibitory factors released by tumor cells or tumor-infiltrating immune cells might mediate such functional impairment of CD3^+^CD56^+^ NKT-like cells, for we observed that tumor tissue culture supernatant from GC patients impaired the effector function of CD3^+^CD56^+^ NKT-like cells. Other studies have shown that tumor microenvironment can secrete inhibitory factor TGF-β1 to prevent effective anti-tumor immune response and blocking TGF-β signaling has emerged as a potential therapeutic approach for tumor treatment [28, 29]. However, our study showed that the functional impairment of CD3^+^CD56^+^ NKT-like cells was not rescued following TGF-β1 blockade, suggesting other soluble factor's involvement from tumor microenvironment rather than TGF-β1.

In conclusion, our study demonstrated that decreased tumor-infiltrating CD3^+^CD56^+^ NKT-like cells and their impaired functionality lead to immune suppression and GC progression, and unveiled that GC microenvironment may form an inhibitory milieu to impair the function of CD3^+^CD56^+^ NKT-like cells. These findings will be helpful for developing novel immune-based GC therapies.

## MATERIALS AND METHODS

### Patients and tissue samples

Fresh peripheral blood, normal autologous gastric tissues (non-tumor, at least 5-cm distance from the tumor site) and tumor tissues were obtained from 63 never-treated GC patients during surgery at the Southwest Hospital of the Third Military Medical University. The clinical stages of tumors were determined according to the TNM classification system of International Union against Cancer (Edition 7). Normal blood was collected from 30 healthy individuals as the control group. The study was approved by the Ethics Committee of the Third Military Medical University, and written informed consent was obtained from each subject. The clinical characteristics of GC Patients were present in Supplementary Table S1.

### Cell isolation

Fresh non-tumor and tumor tissues were used for obtaining cell suspensions as previously described [30]. Briefly, paired non-tumor and tumor tissues were cut into small pieces and collected in RPMI 1640 containing 1mg/ml collagenase IV (Sigma-Aldrich, St.Louis, MO) and 10mg/ml Dnase I (Roche, Basel, Switzerland), then mechanically dissociated by using the gentle MACS Dissociator (Miltenyi Biotec, Auburn, CA). Dissociated cell suspensions were further incubated 1 hour at 37°C under continuous rotation and filtered through 70 μm cell strainers to obtain cell suspensions. The cell suspensions were then used for flow cytometry analysis.

### Flow cytometric analysis

Cells suspensions were stained with appropriate surface antibodies, and then fixed, permeabilized for 20 min using Cytofix/Cytoperm reagent (BD Biosciences), and subsequently stained with antibodies to intracellular molecules granzyme B and Ki-67. For intracellular cytokine staining of IFN-γ and TNF-α, the cells were stimulated for 4 hours with phorbol myristate acetate (PMA, 50 ng/ml) and ionomycin (1 μg/ml) in the presence of Golgistop before staining. The fluorochrome-labeled antibodies are listed in Supplementary Table S2.

### Preparation of tissue culture supernatant

Tumor tissue culture supernatant (TTCS) or non-tumor tissue culture supernatant (NTCS) was prepared as previously described [4].

### Culturing and stimulation of PBMCs

Peripheral blood mononuclear cells (PBMCs) from healthy individuals were isolated by Ficoll density gradient centrifugation. PBMCs were seeded in the 96-well round bottom plates at 4×10^5^ cells/well in RPMI 1640 containing 10% fetal calf serum (Gibco, Bionova, Uruguay). 20 U/ml recombinant human IL-2 (rhIL-2) was supplemented to each well pre-coated with anti-CD3 (2 μg/ml) and anti-CD28 (1 μg/ml) antibodies (Biolegend, San Diego, CA). In some experiments, 10% TTCS or NTCS was also added in the well. Where indicated, anti-TGF-β1 neutralizing antibody (10μg/ml, Abcam) or isotype control IgG antibody was added in the 10% TTCS-treated wells. After 48 hours, plated cells were collected for intracellular granzyme B and Ki-67 expression in CD3^+^CD56^+^ NKT-like cells of PBMCs. For intracellular cytokine staining of IFN-γ and TNF-α, the cells were stimulated at the last 4 hours with PMA (50 ng/ml) and ionomycin (1 μg/ml) in the presence of Golgistop before staining.

### Statistical analysis

All results are summarized as mean ± standard error of the mean (SEM), and statistical analysis was performed with the Prism 5.0 Software. Differences between groups were evaluated by two-tailed Student's *t* test. Cumulative survival time was measured in months and calculated by the Kaplan–Meier method. *P*<0.05 was considered statistically significant.

## SUPPLEMENTARY FIGURES AND TABLES


