# Supplementary material for: Altered phenotypic and functional characteristics of CD3+CD56+ NKT-like cells in human gastric cancer
Source: Oncotarget. 2016 Jul 8;7(34):55222–30. doi: 10.18632/oncotarget.10484 (PMC5342413; doi:10.18632/oncotarget.10484)
Supplement: Supplementary file 1 [file oncotarget-07-55222-s001.pdf]

Altered phenotypic and functional characteristics of CD3⁺CD56⁺ NKT-like cells in human gastric cancer

SUPPLEMENTARY FIGURES AND TABLES

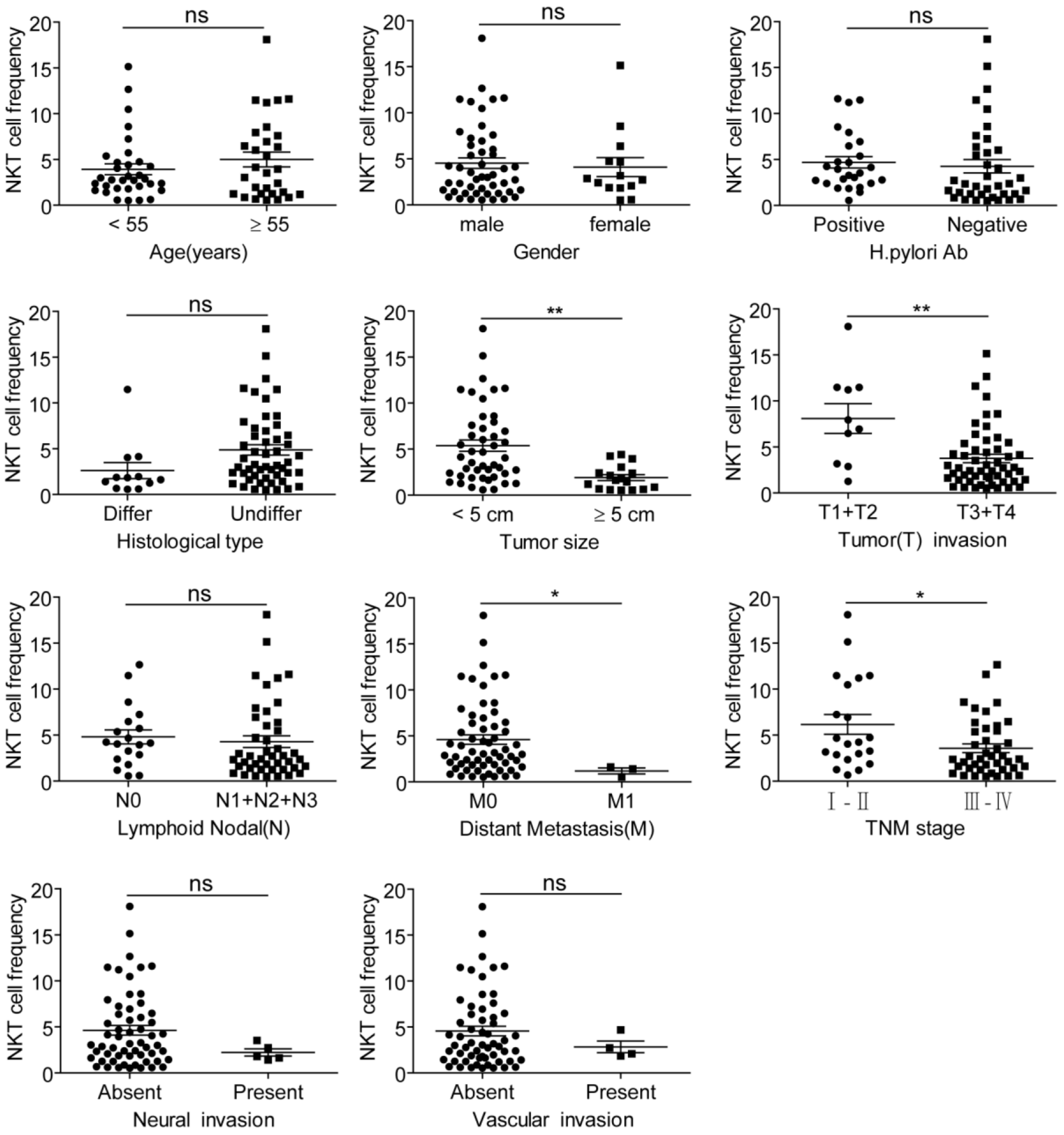

Supplementary Figure S1: CD3+CD56+ NKT-like cell frequency and its potential correlations with clinical parameters. Tumor-infiltrating $CD3^+CD56^+$ NKT-like cell frequency was determined after gating on $CD3^+$ T cells and then analyzed for correlations with clinical parameters. *P<0.05, **P<0.01, ns indicates P>0.05. Each *dot* represents one patient. Ab, antibody; Diff, differentiated; Undiff, undifferentiated.

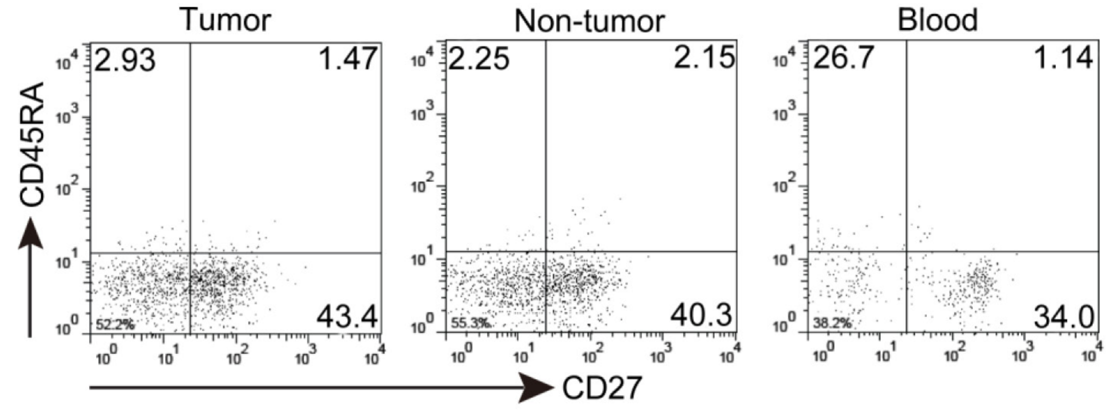

Supplementary Figure S2: A representative flow cytometry analysis of CD45RA and CD27 expression on $CD3^{+}CD56^{+}$ NKT-like cells from tumor tissues, non-tumor tissues and peripheral blood of the same patients (n=4).

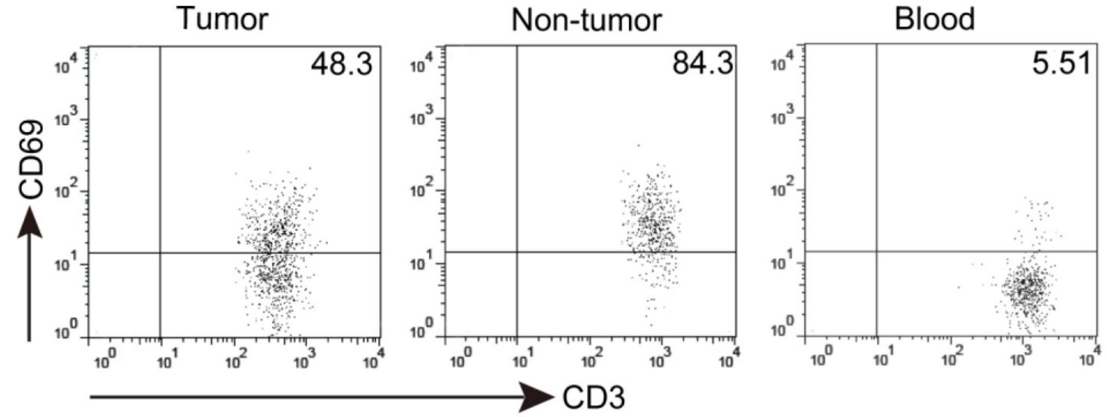

Supplementary Figure S3: A representative flow cytometry analysis of CD69 expression on $CD3^{+}CD56^{+}$ NKT-like cells from paired tumor tissues, non-tumor tissues and peripheral blood (n=5).

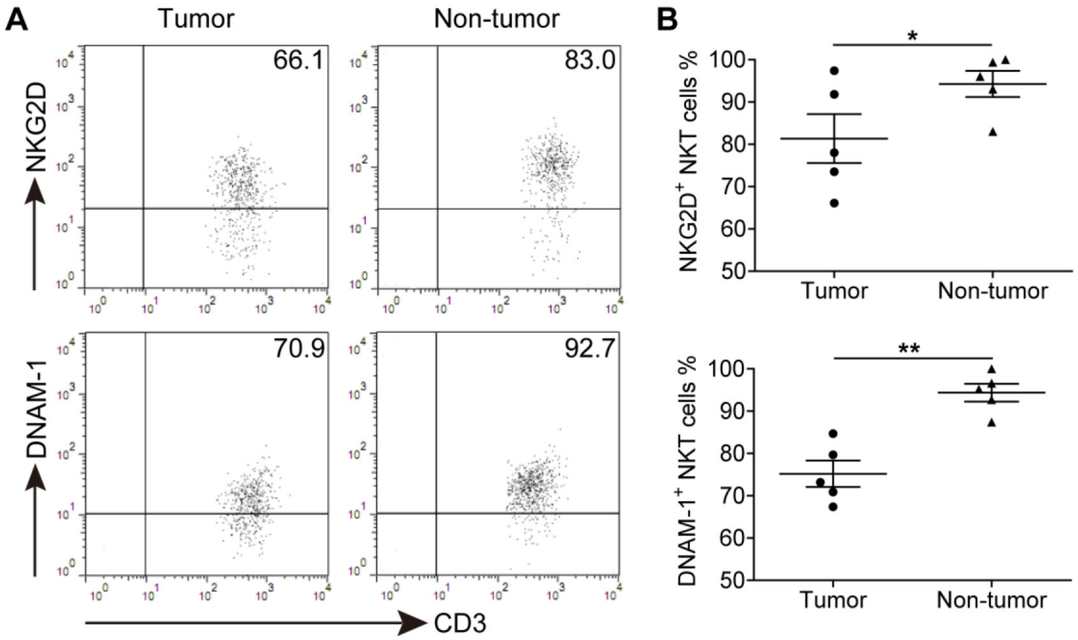

Supplementary Figure S4: A representative flow cytometry A. and Statistical analysis **B.** of NKG2D and DNAM-1 expression on $CD3^{+}CD56^{+}$ NKT-like cells from paired tumor and non-tumor tissues (n=5). *P<0.05, **P<0.01.

Supplementary Table S1: Clinical Characteristics of 63 GC Patients

Variables	No. of patients
Sex (male/female)	49/14
Age (*y*), median (range)	55, 28-82
H pylori antibody (positive/negative)	26/37
Tumor size (cm; <5/≥5)	46/17
Histologic type (differentiated/undifferentiated)	12/51
Neural invasion (absent/present)	58/5
Vascular invasion (absent/present)	59/4
Tumor (T) invasion (T1+T2/T3+T4)	10/53
Lymphoid nodal (N) status (N0 / N1+N2+N3)	19/44
Distant metastasis (M) status (M0/M1)	60/3
TNM stage (I+II/III+IV)	21/42
NK cell percentage (median, range)	2.99, 0.52-18.10

Supplementary Table S2: Fluorochrome-conjugated antibodies used in flow cytometry

Antibodies	Source	Clone
APC-conjugated anti-human CD3	Biolegend	UCHT1
APC-H7 conjugated anti-human CD3	BD Pharmingen	SK7
APC-conjugated anti-human NKp44	Biolegend	P44-8
FITC-conjugated anti-human CD56	Biolegend	HCD56
FITC-conjugated anti-human CD16	Biolegend	3G8
FITC-conjugated anti-human CD45RA	Biolegend	HI100
FITC-conjugated anti-human CD69	Biolegend	FN50
FITC-conjugated anti-human CD62L	Biolegend	DREG-56
FITC-conjugated anti-human CD94	Biolegend	DX22
FITC-conjugated anti-human CD158e1	Biolegend	DX9
FITC-conjugated anti-human CD158a/h	Biolegend	HP-MA4
FITC-conjugated anti-human CXCR3	Biolegend	G025H7
FITC-conjugated anti-human granzyme B	Biolegend	GB11
FITC-conjugated anti-human IFN-γ	Biolegend	B27
FITC-conjugated anti-human DNAM-1	Biolegend	TX25
FITC-conjugated anti-human γδ TCR	Biolegend	B1
FITC-conjugated anti-human Vα 24	Biolegend	6B11
FITC-conjugated anti-human LAG-3	eBioscience	3DS223H
PE-conjugated anti-human CCR5	Biolegend	HEK/1/85a
PE-conjugated anti-human CD27	Biolegend	O323
PE-conjugated anti-human CD38	Biolegend	HB-7
PE-conjugated anti-human CD4	Biolegend	RPA-T4
PE-conjugated anti-human CD57	Biolegend	HNK-1
PE-conjugated anti-human NKp30	Biolegend	P30-15
PE-conjugated anti-human NKG2A	RD systems	131411
PE-conjugated anti-human NKG2D	Biolegend	1D11
PE-conjugated anti-human PD-1	Biolegend	EH12.2H7
PE-conjugated anti-human Tim-3	Biolegend	F38-2E2
PE-conjugated anti-human TIGIT	eBioscience	MBSA43
PE-conjugated anti-human 2B4	Biolegend	C1.7
PE-conjugated anti-human Ki-67	Biolegend	Ki-67
PE-conjugated anti-human TNF-α	Biolegend	MAb11
PE-conjugated anti-human CD158b	Biolegend	DX27
PE-Cy7-conjugated anti-human CD56	Biolegend	MEM-188
PerCP-Cy5.5-conjugated anti-human CD8	Biolegend	RPA-T8
PerCP-Cy5.5-conjugated anti-human HLA-DR	Biolegend	L243
PerCP-Cy5.5-conjugated anti-human NKp46	Biolegend	9E2
